# Draft Genome Sequences of 10 Paenibacillus and Bacillus sp. Strains Isolated from Healthy Tomato Plants and Rhizosphere Soil

**DOI:** 10.1128/MRA.00055-19

**Published:** 2019-03-21

**Authors:** Lu Zhou, Chunxu Song, Anne de Jong, Oscar P. Kuipers

**Affiliations:** a Department of Molecular Genetics, University of Groningen, Groningen, the Netherlands; University of Arizona

## Abstract

In order to investigate the underlying interaction mechanisms between plants and Gram-positive bacteria, 10 Paenibacillus and Bacillus strains were isolated from healthy tomato rhizosphere and plant tissues.

## ANNOUNCEMENT

Tomato is one of the most important horticultural crops in the world. Because of its high nutritional value, tomato fruit ranks first among 40 fruits and vegetables in “relative contribution to human nutrition” (1, 2). However, there are many plant pathogens that can easily infect tomatoes during the growth season and reduce quality and yield (2). In spite of promising results in controlling tomato diseases via chemical treatments, pesticides, and fungicides, residues may cause a big threat to our human health and environment (3). Alternatively, plant growth-promoting Rhizobacteria (PGPR) can promote plant growth as well as inhibit plant pathogen growth, which is an environmentally friendly approach to controlling tomato diseases (4).

Gram-positive bacteria, especially Bacillus and Paenibacillus strains, are among the well-known PGPR strains that can be applied to agriculture to provide biocontrol function (5). In order to elucidate the interaction mechanisms between plant and Paenibacillus and Bacillus species, 10 Paenibacillus- and Bacillus-like strains were isolated from healthy tomato rhizosphere and tissues. Briefly, rhizosphere soil (1 g) of healthy tomato plants was suspended in 9 ml of 10 mM sterilized MgSO_4_ buffer. Then, the suspension was diluted 10^3^ to 10^6^ times with 10 mM sterilized MgSO_4_ buffer. All of the diluted samples were heat treated (80°C) for 15 min and were subsequently spread onto Luria-Bertani (LB) agar plates. The plates were incubated at 28°C for 24 to 48 h to obtain single colonies. For plant tissue isolation, 1 g of tomato leaves was surface sterilized for 1 min in 70% ethanol and for 3 min in 0.5% NaClO solution supplemented with 1 droplet of Tween 80 per 100 ml solution and then was rinsed 5 times with sterilized deionized water. After surface sterilization, the plant tissues were macerated in 9 ml of 10 mM sterilized MgSO_4_ buffer with a sterilized mortar to obtain the plant tissue suspension. The following steps were the same as those for isolation from rhizosphere soil. The surface sterilization process was checked by spreading aliquots of the last rinsing solution on LB agar plates (if no growth was observed after 7 days, surface sterilization was considered to be successful).

A single colony of each strain was grown in 5 ml LB medium at 28°C and 220 rpm. Overnight cultures of the 10 strains in LB medium were collected. Genomic DNA was isolated with a GenElute bacterial genomic DNA kit (Sigma) according to the manufacturer’s protocol. The genomes were sequenced at GATC Biotech (Germany) with an Illumina HiSeq sequencing system. On average, 5 million paired raw reads (150 bp) were generated per sample from each sequencing run and were checked by FastQC version 0.11.5 (6). The low-quality reads were removed using Trimmomatic version 0.38 (7), and the reads were assembled *de novo* using SPAdes version 3.11.1 (8). Default parameters were used for all software unless noted. The coverages of the 10 sequenced genomes all exceeded 150×, and the characteristics of the assemblies and genome features obtained are described in Table 1. The draft genomes were then annotated by the Rapid Annotations using Subsystems Technology (RAST) server (9) and identified to be Paenibacillus or Bacillus by phylogenetic analysis based on the whole-genome sequence of the isolate and other reference genome sequences from NCBI.

**TABLE 1:** Genome features and GenBank accession numbers of the 10 *Paenibacillus* and *Bacillus* strains

Strain^*a*^	Genome size (bp)	G+C content (%)	No. of coding sequences	*N*_50_ (bp)	No. of contigs	GenBank accession no.	SRA accession no.
*Bacillus subtilis* BH5	4,140,601	44.0	4,221	997,181	29	RPHI00000000	SRR8443430
*Bacillus subtilis* BH6	4,139,877	44.0	4,224	997,721	28	RPHC00000000	SRR8443431
*Bacillus subtilis* DH12	4,180,980	43.3	4,329	1,062,805	27	RQPH00000000	SRR8443428
*Bacillus subtilis* EH2	4,125,144	43.5	4,327	1,048,476	23	RPHG00000000	SRR8443427
*Bacillus subtilis* EH5	4,157,573	43.5	4,352	1,073,629	21	RPHF00000000	SRR8443424
*Bacillus subtilis* EH11	4,179,885	43.3	4,335	1,062,805	26	RPHE00000000	SRR8443426
*Bacillus endophyticus* FH5	5,366,783	36.4	5,462	351,654	53	RPHD00000000	SRR8443432
*Bacillus velezensis* FH17	4,280,415	45.7	4,408	362,129	29	RQPG00000000	SRR8443425
*Bacillus velezensis* TH16	3,952,155	46.4	3,975	298,227	43	RQPF00000000	SRR8443433
*Paenibacillus xylanexedens* EDO6	7,354,453	45.6	6,553	1,358,350	26	RPHH00000000	SRR8443429

a Paenibacillus xylanexedens EDO6 was isolated from tomato plant leaves; the other nine strains were isolated from tomato plant rhizosphere soil.

### Data availability.

The draft genome sequences of the 10 strains have been deposited in GenBank under the accession numbers listed in Table 1. The raw reads have been registered and submitted to the Sequence Read Archive (SRA) under the accession numbers listed in Table 1.
